# Improvement of DC Performance and RF Characteristics in GaN-Based HEMTs Using SiN_x_ Stress-Engineering Technique

**DOI:** 10.3390/nano14181471

**Published:** 2024-09-10

**Authors:** Chenkai Deng, Peiran Wang, Chuying Tang, Qiaoyu Hu, Fangzhou Du, Yang Jiang, Yi Zhang, Mujun Li, Zilong Xiong, Xiaohui Wang, Kangyao Wen, Wenmao Li, Nick Tao, Qing Wang, Hongyu Yu

**Affiliations:** 1 School of Energy Science and Engineering, Harbin Institute of Technology, Harbin 150001, China; 12149033@mail.sustech.edu.cn (C.D.); 12049024@mail.sustech.edu.cn (C.T.); liwm@mail.sustech.edu.cn (W.L.); 2 School of Microelectronics, Southern University of Science and Technology, Shenzhen 518055, China; 12333281@mail.sustech.edu.cn (P.W.); 12231180@mail.sustech.edu.cn (Q.H.); 11811803@mail.sustech.edu.cn (F.D.); 12132453@mail.sustech.edu.cn (M.L.); 12132480@mail.sustech.edu.cn (Z.X.); 12231174@mail.sustech.edu.cn (X.W.); 3 Faculty of Engineering, The University of Hong Kong, Hong Kong 999077, China; 11510044@mail.sustech.edu.cn (Y.J.); zhangyi97@connect.hku.hk (Y.Z.); 4 State Key Laboratory of ASIC and System, School of Microelectronics, Fudan University, Shanghai 200433, China; 22112020122@m.fudan.edu.cn; 5 Maxscend Microelectronics Company Ltd., Wuxi 214072, China; nick.tao@maxscend.com; 6 Engineering Research Center of Integrated Circuits for Next-Generation Communications, Ministry of Education, Southern University of Science and Technology, Shenzhen 518055, China

**Keywords:** GaN HEMTs, RF, gate leakage, SiN_x_ stress-engineered

## Abstract

In this work, the DC performance and RF characteristics of GaN-based high-electron-mobility transistors (HEMTs) using the SiN_x_ stress-engineered technique were systematically investigated. It was observed that a significant reduction in the peak electric field and an increase in the effective barrier thickness in the devices with compressive SiN_x_ passivation contributed to the suppression of Fowler–Nordheim (FN) tunneling. As a result, the gate leakage decreased by more than an order of magnitude, and the breakdown voltage (BV) increased from 44 V to 84 V. Moreover, benefiting from enhanced gate control capability, the devices with compressive stress SiN_x_ passivation showed improved peak transconductance from 315 mS/mm to 366 mS/mm, along with a higher cutoff frequency (*f*_t_) and maximum oscillation frequency (*f*_max_) of 21.15 GHz and 35.66 GHz, respectively. Due to its enhanced frequency performance and improved pinch-off characteristics, the power performance of the devices with compressive stress SiN_x_ passivation was markedly superior to that of the devices with stress-free SiN_x_ passivation. These results confirm the substantial potential of the SiN_x_ stress-engineered technique for high-frequency and high-output power applications, which are crucial for future communication systems.

## 1. Introduction

GaN-based high-electron-mobility transistors (HEMTs) are considered promising for high-frequency and high-power applications due to the excellent properties of their wide-bandgap semiconductor materials, such as a wide bandgap, high critical breakdown electric field, and high electron saturation velocity [1,2,3]. Current collapse and gate leakage are key reliability challenges for GaN RF devices. A silicon nitride (SiN_x_) film grown by plasma-enhanced chemical vapor deposition (PECVD) is the most commonly used passivation layer to mitigate current collapse [4,5]. However, this process is often accompanied by a detrimental rise in the gate leakage current [6,7], which leads to breakdown voltage, power-added efficiency (PAE), and output power degradation [8].

Most researchers believe that the “virtual gate” effect caused by surface traps enhances the electric field on the drain side of the gate edge, resulting in increased gate leakage current in PECVD-SiN_x_-passivated GaN HEMTs [9]. Additionally, some researchers believe that the active plasma source used in the PECVD process can damage the (Al)GaN surface and the deposited film itself, leading to surface traps, surface erosion, or dangling bond defects [10]. Consequently, this results in poor passivation protection and increased leakage current. Due to the inevitable plasma damage caused by traditional PECVD SiN_x_ passivation, the MOCVD [11], remote ICP-CVD [12], and LPCVD [13] techniques have been proposed for non-destructive passivation of GaN HEMTs to achieve lower gate leakage. Furthermore, the use of N_2_O plasma remote treatment [14] or deposition of a thin layer of Al metal [15] before PECVD passivation can effectively mitigate plasma source bombardment on GaN HEMTs’ surfaces, thereby enhancing pinch-off characteristics. 

In this work, we propose the SiN_x_ stress-engineering technique as a novel and straightforward method to reduce gate leakage while enhancing breakdown voltage (BV), transconductance (*g*_m_), saturation output current, cutoff frequency (*f*_t_), maximum oscillation frequency (*f*_max_), and power performance. These improvements are akin to the performance gains seen in early-strained silicon CMOS technologies, which demonstrated significant scaling and performance enhancements [16]. The reduction in gate leakage is primarily attributed to SiN_x_ stress passivation, which effectively lowers the peak electric field and increases the effective barrier thickness of AlGaN, thereby suppressing Fowler–Nordheim (FN) tunneling. Additionally, the device’s BV characteristics have nearly doubled, saturation output current has increased by 10%, *g*_m_ has improved from 315 mS/mm to 366 mS/mm, and both *f*_t_ and *f*_max_ have shown significant enhancements. Due to enhanced frequency performance, maximized output current, and improved pinch-off characteristics, devices with compressive stress SiN_x_ passivation demonstrate superior output power (*P*_out_), power-added efficiency (PAE), and associated gain.

## 2. Device Structure and Fabrication Process

**Figure 1:**
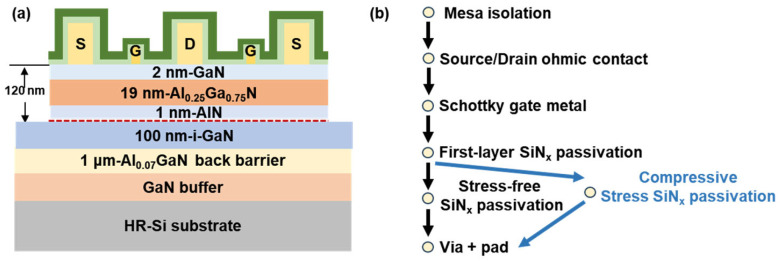
(**a**) Schematic diagram and (**b**) process flow of AlGaN/GaN-on-Si HEMTs with stress-free SiN_x_ passivation and compressive stress SiN_x_ passivation.

The epitaxial structure of the AlGaN/GaN HEMTs in this work is shown in Figure 1a. The 6-inch Si wafer with MOCVD-grown GaN/Al_0.25_Ga_0.75_N/AlN/GaN epitaxy is purchased from Enkris Semiconductor. The epilayer, from bottom to top, consists of a 1.05 μm high-resistivity (Al)GaN buffer layer, a 1 μm Al_0.07_GaN back barrier layer, a 100 nm unintentionally doped i-GaN channel layer, a 1 nm AlN spacer, a 19 nm Al_0.25_Ga_0.75_N barrier layer, and a 2 nm GaN cap layer. Room-temperature Hall effect measurements indicated a sheet carrier density (*n*_s_) of 7.23 × 10^12^ cm^−2^, an electron mobility (μ) of 2051 cm^2^/(V·s), and a sheet resistance (*R*_sh_) of 400 Ω/□.

**Figure 2:**
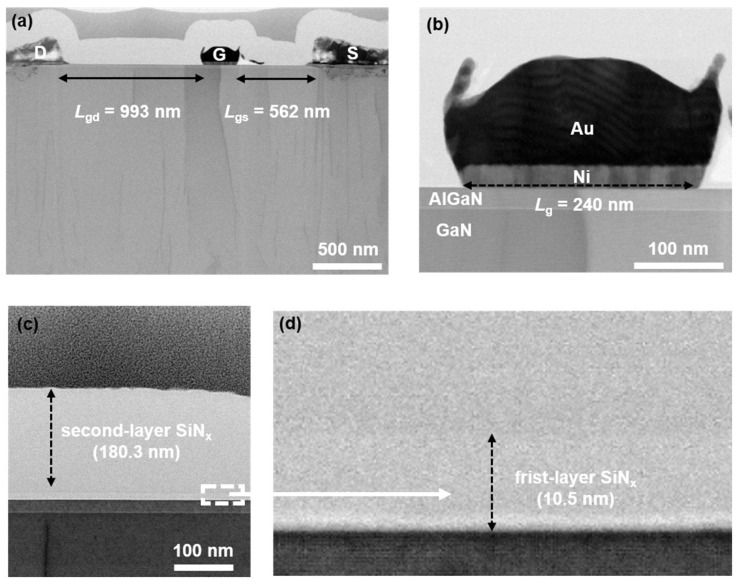
(**a**) SEM images of overall device. TEM images of (**b**) gate metal stack (**c**,**d**) PECVD dual-layer SiN_x_, composed of a 10.5 nm SiN_x_ protection layer and a 180.3 nm SiN_x_ stress layer.

As shown in Figure 1b, the device fabrication process begins with device isolation using BCl_3_/Cl_2_-based inductively coupled plasma (ICP) dry etching. This is followed by depositing a Ti/Al/Ti/Au (20/110/40/50 nm) metal stack using an e-beam evaporator (e-beam) and annealing at 830 °C for 45 s under ambient nitrogen in a rapid thermal annealing (RTA) system to form the source/drain ohmic contacts. The gate region is then patterned using electron-beam lithography (EBL) with polymethyl methacrylate (PMMA), and the Ni/Au (20/60 nm) metal gate is fabricated using an e-beam evaporator. The SiN_x_ layers were deposited by PECVD with dual plasma excitation frequencies using silane (SiH_4_) and ammonia (NH_3_) as precursors. Subsequently, Ti/Au (20/180 nm) metal pads were deposited after CHF_3_-based opening. Figure 2a,b show the SEM images of the overall device and the TEM images of the gate region of the fabricated GaN RF device, along with the measured device dimensions. The reported devices feature a gate length (*L*_g_) of 0.24 μm, a gate width (*W*_g_) of 2 × 25 μm, a gate–drain length (*L*_gd_) of 993 nm, and a gate–source length (*L*_gs_) of 562 nm.

**Figure 3:**
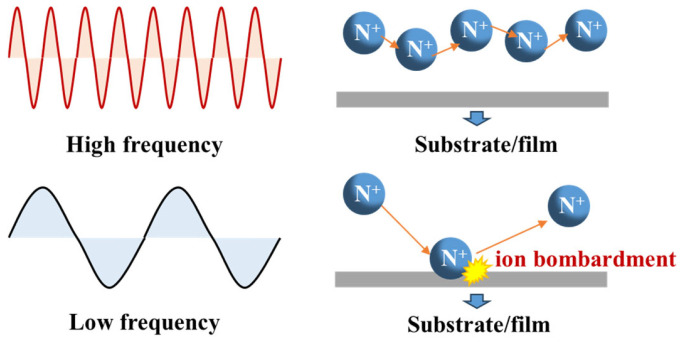
Schematics for the nitrogen ions responding to different plasma excitation frequencies in PECVD.

To investigate the impact of stress on the DC and RF characteristics of GaN RF devices, we fabricated two types of devices with different stress SiN_x_ passivation layers. As shown in Table 1, all devices feature a double-layer passivation structure. The first layer is a ~10 nm high-frequency (HF) SiN_x_ protection layer with a refractive index of 2.13, designed to minimize surface damage, while the second layer is ~180 nm SiN_x_ stress. To modify the intrinsic stress of the PECVD SiN_x_ layers, several deposition parameters can be adjusted, including the Si ratio, chamber pressure, deposition temperature, and plasma excitation frequency [17]. Specifically, for PECVD systems utilizing dual plasma excitation frequencies, adjusting the duty cycles of the high-frequency (HF) and low-frequency (LF) RF power sources allows for a broad modulation of the intrinsic stress of the deposited SiN_x_. During HF excitation (e.g., 13.56 MHz), the ions do not respond significantly to the RF field, leading to the formation of low-stress SiN_x_ films. Conversely, under LF excitation (e.g., 500 kHz), ions are more responsive to the RF field, resulting in ion bombardment on the growing SiN_x_ film. This ion bombardment, as illustrated in Figure 3, densifies the film and causes it to expand against its inherent volume, thereby inducing intrinsic compressive stress [17].

**Figure 4:**
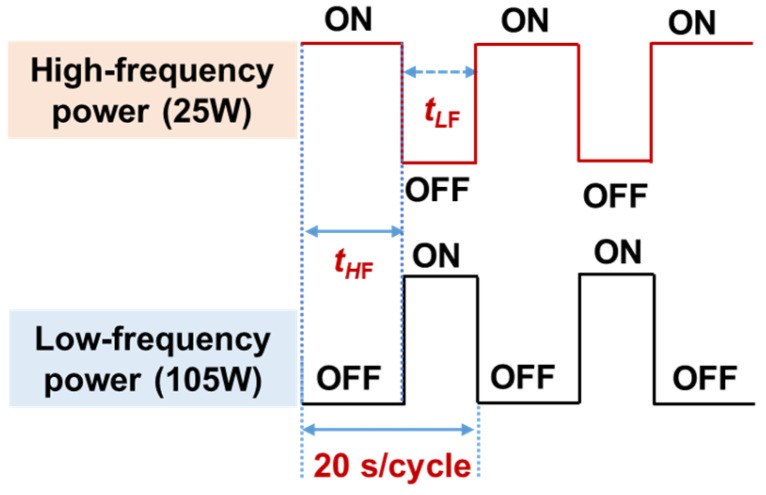
Intrinsic stress of PECVD SiN_x_ can be modulated by adjusting the duty cycle of the low-frequency (LF) plasma excitation.

As shown in Figure 4, adjusting the duty cycles of LF plasma excitation modulates the intrinsic stress of SiN_x_. In this work, the devices with a SiN_x_ stress layer of 45% with a refractive index of 2.04 and 95% LF duty cycle with a refractive index of 1.95 correspond to stress-free SiN_x_ passivation and compressive SiN_x_ stress passivation, respectively. In our previous work, we utilized Raman spectroscopy to confirm the existence the level of stress within the AlGaN/GaN heterostructure covered by different SiN_x_ layers [18]. After the two different SiN_x_ depositions, we extracted the *R*_sh_ of the devices using the transmission line model (TLM). Since both samples employed the same HF SiN_x_ passivation process for the first layer, the effectiveness in suppressing surface states was consistent. As a result, the sheet resistance values were 384 Ω/□ and 379 Ω/□ for devices with stress-free SiN_x_ and compressive stress SiN_x_ passivation, respectively, both lower than the initial value of 400 Ω/□. The observed decrease in sheet resistance is primarily due to the increased 2DEG density achieved through SiN_x_ passivation.

## 3. Results and Discussion

**Figure 5:**
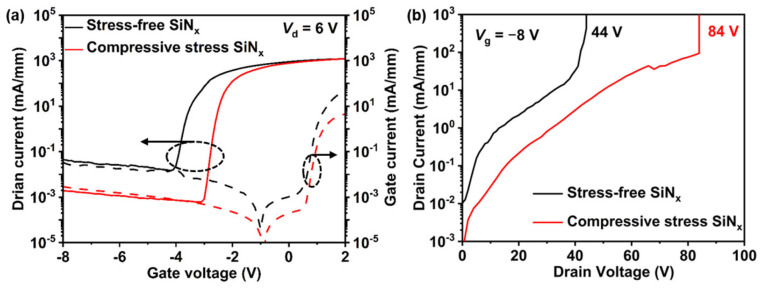
(**a**) The transfer characteristics when *V*_ds_ = 6 V of devices with stress-free SiN_x_ passivation and compressive stress SiN_x_ passivation. (**b**) The *I*_d_/*V*_d_ curve when *V*_g_ = −8 V of the device with stress-free SiNx passivation and compressive stress SiN_x_ passivation.

A Keithley 4200 semiconductor parameter analyzer (Tektronix, Beaverton, OR, USA) was used for DC measurements. Figure 5a shows the transfer characteristics of each GaN HEMT when *V*_ds_ = 6 V. Due to the SiN_x_ compressive stress depleting the 2DEG under the gate region by neutralizing the original piezoelectric polarization, the devices show a 1 V increase in threshold voltage (*V*_th_) compared to those devices with stress-free SiN_x_ passivation. The reasons for the positive shift in threshold voltage have been discussed in detail in our previous work [19]. Moreover, devices with compressive stress SiN_x_ passivation demonstrate more than an order of magnitude reduction in leakage current. The BV of the devices was also significantly improved from 44 V to 84 V, as shown in Figure 5b.

The reverse gate leakage current is predominantly attributed to Poole–Frenkel (PF) emission and FN tunneling mechanisms [10,11,12,13,14,15,16,17,18,19,20,21]. PF emission is the dominant leakage mechanism for structures with lower mole fractions. When the Al composition exceeds 0.25, the gate leakage current is primarily dominated by FN tunneling, as reported in the literature [22]. The prominence of the FN tunneling component at room temperature and above in higher mole fraction structures is attributed to the higher electric field resulting from increased values of net bound charge (*σ*_b_) [22]. The dependence of FN tunneling current density (*J_FN_*) on the barrier electric field (*E*) is given by

(1) $$J_{FN}=AE^{2}e^{\left(\frac{-B}{E}\right)}$$

where *J* is the tunneling current density, *E* is the electric field strength, and A and B are constants related to the material and barrier properties.

(2) $$A=\frac{q^{3}E^{2}}{8\pi h\phi_{b}}$$

**Figure 6:**
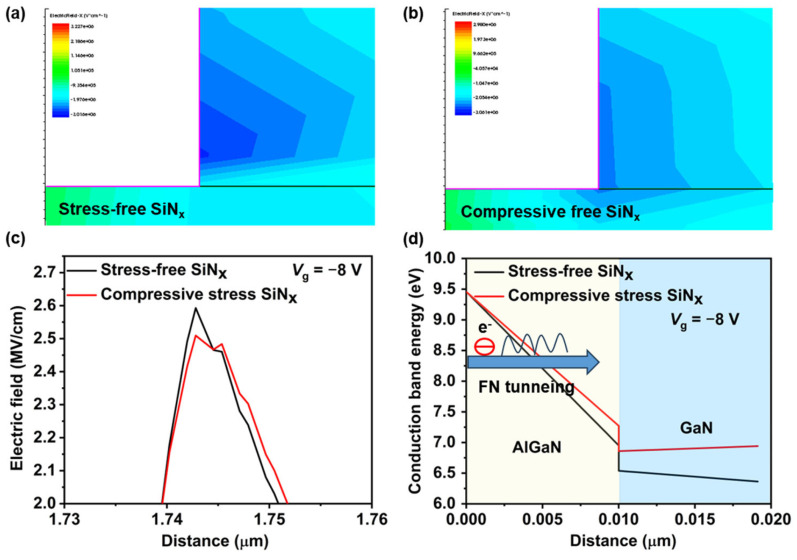
The electric field distribution near the gate–drain side of devices with (**a**) stress-free SiN_x_ passivation and (**b**) compressive stress SiN_x_ passivation. (**c**) The electric field value comparison near the gate–drain, and (**d**) conduction band diagram when *V*_g_ = −8 V of the devices with stress-free SiN_x_ passivation and compressive stress SiN_x_ passivation.

(3) B=−8π2mϕb323hq

where *q* is the electron charge, *h* is Planck’s constant, *m* is the electron mass, *ϕ*_b_ is the effective barrier height, and *E* is the electric field strength. To explore the intrinsic mechanism of the stress-engineered technique in suppressing FN tunneling, we utilized technology computer-aided design (TCAD) Sentaurus to simulate the electric field distribution and conduction band diagram of those devices with stress-free SiN_x_ passivation and compressive stress SiN_x_ passivation with the model parameters calibrated. As shown in Figure 6a,b the introduction of compression neutralizes the inherent piezoelectric polarization caused by lattice mismatch at the heterojunction, leading to a significant reduction in the peak electric field in the gate region. Figure 6c extracts the electric field values near the gate–drain side; the devices with compressive stress SiN_x_ passivation show a 0.1 MV/cm decrease compared to devices with stress-free SiN_x_ passivation. The conduction band diagrams in the gate region of devices when *V*_g_ = −8 V are shown in Figure 6d. The external compressive stress liner elevates the conduction bands in the AlGaN barrier and GaN channel, thereby reducing the slope of the AlGaN conduction band and effectively increasing the effective barrier thickness. As a result, FN tunneling is suppressed in devices with compressive stress SiN_x_ passivation, reducing the gate leakage. Additionally, the breakdown voltage of the device has correspondingly improved, as shown in Figure 4b.

**Figure 7:**
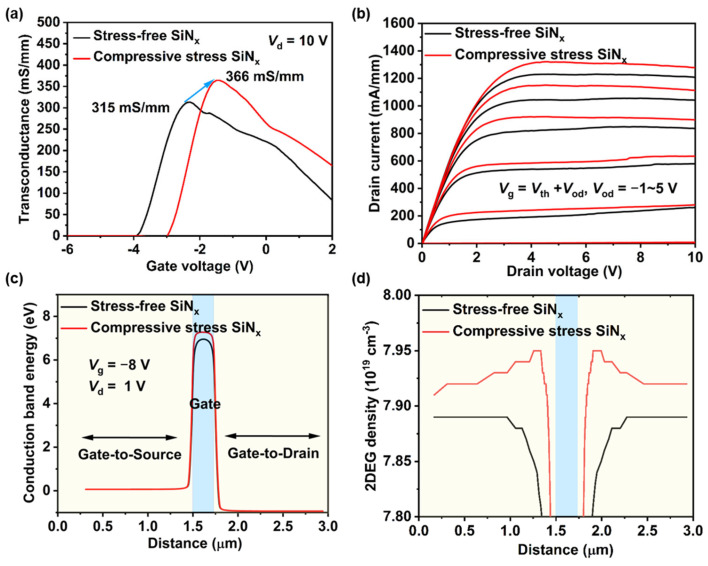
(**a**) The transconductance curves, (**b**) the output characteristics when override voltage (*V*_od_) = −1 to 5 V, (**c**) the conduction band energy of AlGaN beneath the gate, and (**d**) 2DEG concentration distribution of the devices with stress-free SiN_x_ passivation and compressive stress SiN_x_ passivation.

Figure 7a illustrates the transconductance curves of those devices, the devices with compressive stress SiN_x_ passivation exhibit a significant improvement in the extrinsic peak transconductance (*g*_m,max_), from 315 mS/mm to 366 mS/mm. This enhancement primarily stems from the improved conduction band of AlGaN beneath the gate of GaN HEMTs due to SiN_x_ stress engineering, thereby enhancing gate modulation capability, as depicted in Figure 7c. The output characteristics when override voltage (*V*_od_) = −1 to 5 V are shown in Figure 7b. The maximum drain current density (*I*_d,max_) of devices with compressive stress SiN_x_ passivation also shows a notable enhancement. The improved drain current was supposedly due to the SiN_x_ stressors causing tensile stress in the gate–drain and gate–source regions, inducing more channel 2DEG, as shown in Figure 7d.

**Figure 8:**
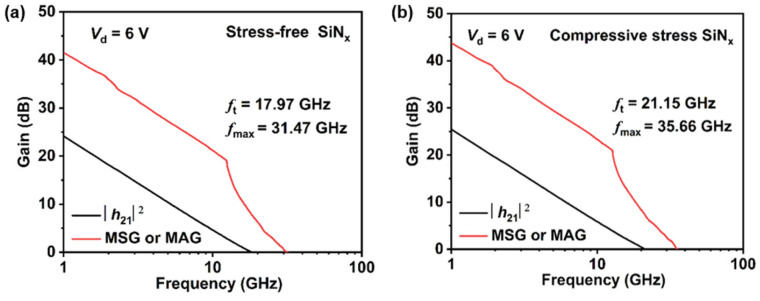
Small-signal performance biased at *V*_ds_ = 6 V and their respective *V*_g_ for the *g*_m,max_ of the devices (**a**) with stress-free SiN_x_ passivation and (**b**) compressive stress SiN_x_ passivation.

S-parameters were measured using an Agilent 8363B network analyzer (Agilent, Santa Clara, CA, USA). The small-signal performances of the GaN-based HEMTs with stress-free SiN_x_ passivation and compressive stress SiN_x_ passivation are illustrated in Figure 8a,b, with the devices biased at *V*_ds_ = 6 V to obtain their respective *V*_g_ for the *g*_m,max_. Due to the improved transconductance, the devices with compressive stress SiN_x_ passivation exhibited higher *f*_t_ and *f*_max_, measured as 21.15 GHz and 35.66 GHz, respectively.

**Figure 9:**
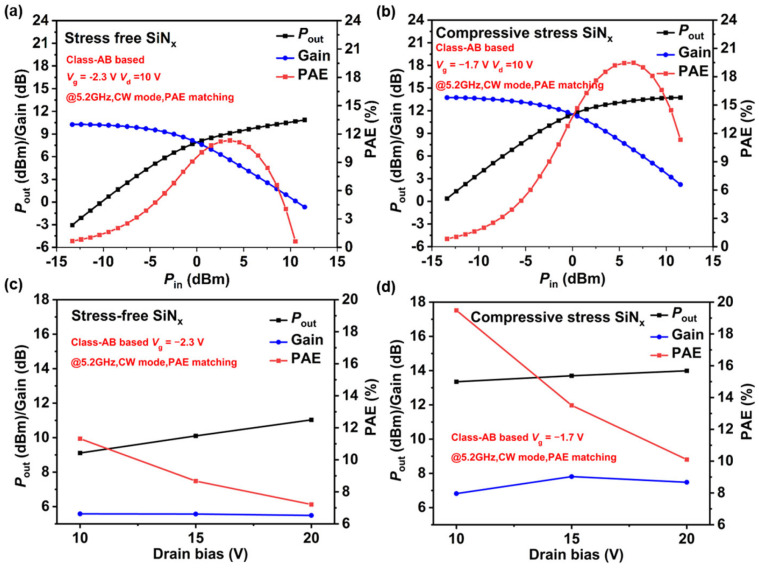
CW power performance at bias of *V*_ds_ = 10 V of GaN HEMTs (**a**) with stress-free SiN_x_ passivation and (**b**) compressive stress SiN_x_ passivation. Measured output power density, PAE, and associated gain versus drain bias at 5.2 GHz of GaN HEMTs (**c**) with stress-free SiN_x_ passivation and (**d**) compressive stress SiN_x_ passivation.

Power measurements of AlGaN/GaN HEMTs at 5.2 GHz were conducted in continuous wave (CW) mode using an on-wafer load-pull system. The load and source impedances were tuned for optimal PAE, which led to a slightly lower power gain compared to that observed in the small-signal performance. Figure 9a,b show the output power, power gain, and PAE as a function of the input power for the devices with stress-free SiN_x_ passivation and compressive stress SiN_x_ passivation. A maximum *P*_out_ of 13.35 dBm, along with a PAE of 19.48% and an associated gain of 6.82 dB, is achieved for the devices with compressive stress SiN_x_ passivation when biased at *V*_ds_ = 10 V. Figure 9c,d illustrate the impact of drain bias on the device’s output power, PAE, and associated gain, with all measurements conducted under Class AB operation. Regardless of the drain bias, the power performance of the devices with compressive stress SiN_x_ passivation is markedly superior to that of the devices with stress-free SiN_x_ passivation, which is attributed to its enhanced frequency performance, maximized output current, and improved pinch-off characteristics.

## 4. Conclusions

In summary, this study investigated the DC performance and RF characteristics of GaN-based HEMTs using the SiN_x_ stress-engineering technique. Devices with compressive stress SiN_x_ passivation exhibited a significant reduction in peak electric field and an increase in effective barrier thickness, effectively suppressing FN tunneling. Consequently, there was a substantial reduction in gate leakage and an increase in breakdown voltage (BV) from 44 V to 84 V. Furthermore, enhanced gate control capability led to an improvement in peak transconductance, increasing from 315 mS/mm to 366 mS/mm, along with a higher cutoff frequency (*f*_t_) and maximum oscillation frequency (*f*_max_) of 21.15 GHz and 35.66 GHz, respectively. Due to the SiN_x_ stressors causing tensile stress in the gate–drain and gate–source regions, which induced more channel 2DEG, the device’s saturation current also increased by 10%. The power performance of the devices with compressive stress SiN_x_ passivation was also markedly superior to that of devices with stress-free SiN_x_ passivation, attributed to enhanced frequency performance, maximized output current, and improved pinch-off characteristics. These results indicate that the SiN_x_ stress-engineering technique is a potentially effective approach for achieving high-performance GaN-on-Si HEMTs for RF electronics applications.

## Figures and Tables

**Table 1:** SiN_x_ schemes for device groups.

Passivation Scheme		Stress-Free SiN_x_ Passivation	Compressive SiN_x_ Passivation
First-layer SiN_x_ passivation		10 nm high-frequency SiN_x_ protective layer (t_LF_/20 = 0)	
Second-layer SiN_x_ passivation	Thickness	180 nm	180 nm
	LF duty cycle	t_LF_/20 = 45%	t_LF_/20 = 95%

## Data Availability

The data that support the findings of this study are available from the corresponding authors upon reasonable request.
